# Remifentanil discontinuation and subsequent intensive care unit-acquired infection: a cohort study

**DOI:** 10.1186/cc7788

**Published:** 2009-04-21

**Authors:** Saad Nseir, Jérémy Hoel, Guillaume Grailles, Aude Soury-Lavergne, Christophe Di Pompeo, Daniel Mathieu, Alain Durocher

**Affiliations:** 1 Intensive Care Unit, Calmette Hospital, University Hospital of Lille, boulevard du Pr Leclercq, 59037 Lille cedex, France; 2 Medical Assessment Laboratory, Lille II University, 1 place de Verdun, 59045 Lille, France

## Abstract

**Introduction:**

Recent animal studies demonstrated immunosuppressive effects of opioid withdrawal resulting in a higher risk of infection. The aim of this study was to determine the impact of remifentanil discontinuation on intensive care unit (ICU)-acquired infection.

**Methods:**

This was a prospective observational cohort study performed in a 30-bed medical and surgical university ICU, during a one-year period. All patients hospitalised in the ICU for more than 48 hours were eligible. Sedation was based on a written protocol including remifentanil with or without midazolam. Ramsay score was used to evaluate consciousness. The bedside nurse adjusted sedative infusion to obtain the target Ramsay score. Univariate and multivariate analyses were performed to determine risk factors for ICU-acquired infection.

**Results:**

Five hundred and eighty-seven consecutive patients were included in the study. A microbiologically confirmed ICU-acquired infection was diagnosed in 233 (39%) patients. Incidence rate of ICU-acquired infection was 38 per 1000 ICU-days. Ventilator-associated pneumonia was the most frequently diagnosed ICU-acquired infection (23% of study patients). *Pseudomonas aeruginosa *was the most frequently isolated microorganism (30%). Multivariate analysis identified remifentanil discontinuation (odds ratio (OR) = 2.53, 95% confidence interval (CI) = 1.28 to 4.99, *P *= 0.007), simplified acute physiology score II at ICU admission (1.01 per point, 95% CI = 1 to 1.03, *P *= 0.011), mechanical ventilation (4.49, 95% CI = 1.52 to 13.2, *P *= 0.006), tracheostomy (2.25, 95% CI = 1.13 to 4.48, *P *= 0.021), central venous catheter (2.9, 95% CI = 1.08 to 7.74, *P *= 0.033) and length of hospital stay (1.05 per day, 95% CI = 1.03 to 1.08, *P *< 0.001) as independent risk factors for ICU-acquired infection.

**Conclusions:**

Remifentanil discontinuation is independently associated with ICU-acquired infection.

## Introduction

Healthcare-associated infections are the most common complications affecting hospitalised patients [1]. Intensive care unit (ICU)-acquired infections represent the major part of these infections [2]. In a recent multicentre study conducted in 71 adult ICUs [3], 7.4% of the 9493 included patients had an ICU-acquired infection. ICU-acquired pneumonia (47%) and ICU-acquired bloodstream infection (BSI) (37%) were the most frequently reported infections. Another recent multicentre study was conducted in 189 ICUs [4]. Of the 3147 included patients, 12% had an ICU-acquired sepsis. ICU-acquired infections are frequently advocated as a significant contributor to mortality and morbidity [5,6]. Identifying risk factor for healthcare-associated infections could be helpful for future studies aiming at preventing these infections.

Sedative and analgesic medications are routinely used in mechanically ventilated patients to reduce pain and anxiety and to allow patients to tolerate invasive procedures in the ICU [7]. Mostly a combination of an opioid, to provide analgesia, and a hypnotic, such as benzodiazepines or propofol to provide anxiolysis, is used. A variety of opioids used by intravenous administration in adults are available for use in the ICU, including morphine, fentanyl, alfentanil, sufentanil and remifentanil [8]. The use of fentanil, alfentanil and sufentanil, as well as morphine, is always accompanied by concerns regarding drug accumulation. In contrast, remifentanil is a short-acting opioid which is characterised by a rapid and uniform clearance and a highly predictable onset and offset of effect [9]. Recently, negative effects of opioid withdrawal on the immune system were reported in opiate abusers [10,11]. In addition, animal studies suggested that morphine withdrawal induces immunosuppression resulting in an increased risk of infection [12,13]. We hypothesised that remifentanil discontinuation would be associated with a higher risk for subsequent ICU-acquired infection.

## Materials and methods

### Study design

This prospective observational cohort study was conducted in a 30-bed medical and surgical university ICU from December 2006 to December 2007. In accordance with the French law, approval by the local Institutional Review Board and informed consent was not required, given that this observational study did not modify current diagnostic or therapeutic strategies. All patients hospitalised in the ICU for more than 48 hours were eligible for this study.

### Study population

The infection control policy included isolation techniques, routine screening of multidrug-resistant (MDR) bacteria, written antibiotic treatment protocol and continuous surveillance of nosocomial infections. Isolation techniques were performed in all patients with colonisation or infection related to MDR bacteria and in all immunosuppressed patients [14]. These techniques included protective gowns and gloves usage associated with adequate hand hygiene using alcohol-based hand rub formulation before and after patient contacts. No selective digestive decontamination was performed.

### Sedation protocol

Sedation was based on a written protocol including remifentanil with or without midazolam. Ramsay score was used to evaluate consciousness [15]. The target Ramsay score was determined by the physicians. The bedside nurse adjusted sedative infusion to obtain target sedation level. Remifentanil was first used to obtain the sedation target and infusion of remifentanil could be increased every five minutes. If maximal dose of remifentanil was insufficient to obtain the prescribed Ramsay score, midazolam infusion was started and adjusted by the bed-side nurse. For example, in a patient weighing 60 kg, midazolam was started if remifentanil was insufficient to obtain the target Ramsay score at 58 mg/day. No daily interruption of sedation was performed. Sedation discontinuation was at the physician's discretion. However, in patients who received a combination of remifentanil and midazolam, midazolam was always discontinued before or at the same time as remifentanil. Acute withdrawal was treated by levomepronazin and lorazepam. Propofol infusion was used to treat acute withdrawal refractory to these medications.

### Data collection

All data were prospectively collected. At ICU admission, the following data were collected: age; gender; simplified acute physiology score (SAPS) II [16]; logistic organ dysfunction score [16]; McCabe score; admission category; presence of comorbidities, including chronic obstructive pulmonary disease [17], chronic heart failure, immunosuppression [14] and diabetes mellitus; presence of infection; prior antibiotic treatment; and length of hospital stay before ICU admission. During ICU stay, the following data were collected: central venous and arterial catheter use; urinary tract catheter use; mechanical ventilation; duration of use of catheters and mechanical ventilation; reintubation; tracheostomy; fibreoptic bronchoscopy; digestive tract endoscopy; antimicrobial treatment; duration of antimicrobial treatment; remifentanil and midazolam use; duration and doses of remifentanil and midazolam used; discontinuation of remifentanil and midazolam; neuromuscular blocking agent use; and acute withdrawal. Information on length of ICU stay and ICU mortality was also collected.

### Definitions

Sedation discontinuation was defined as an interruption of all sedatives for at least 24 hours, except those medications given to treat acute withdrawal. Acute withdrawal was defined by the presence of at least five of the following criteria [18] during the six hours following sedation discontinuation: fever (> 38°C), tachycardia (> 100 beats/minute), hypertension (mean arterial pressure > 100 mmHg), sweating, mydriasis, diarrhoea, nausea/vomiting and restlessness. Infection was considered as ICU-acquired if it was diagnosed more than 48 hours after ICU admission. ICU-acquired infections occurring less than five days after ICU admission were considered as early onset. Late-onset ICU-acquired infections were defined as those infections diagnosed five days or more after ICU admission.

Ventilator-associated pneumonia (VAP) was defined by the presence of new or progressive radiographic infiltrate associated with two of the following criteria: temperature above 38.5°C or below 36.5°C; leukocyte count greater than 10000 cells/μL or less than 1500 cells/μl; purulent tracheal aspirate. In addition, a positive tracheal aspirate (≥ 10^6 ^colony-forming units (cfu)/ml); or bronchoalveolar lavage (≥ 10^4 ^cfu/ml) was required [19].

BSI was defined as onset of infection associated with one or more positive blood culture result unrelated to an infection incubating at ICU admission. Coagulase-negative *Staphylococcus *BSI was defined as two or more positive blood cultures on separate occasions within a 48-hour period, or at least one blood culture positive with clinical sepsis, no other infectious process and antibiotic treatment given by the attending physician [20]. Ventilator-associated tracheobronchitis was defined by all of the following criteria: fever (>38°C) with no other recognisable cause, purulent sputum production, positive (≥ 10^6 ^cfu/ml) endotracheal aspirate culture and no radiographic signs of new pneumonia [21]. All Other ICU-acquired infections were defined according to modified Centers for Disease Control and Prevention criteria [22]. Only infections confirmed by microbiological results were taken into account.

Incidence rate was defined as the number of ICU-acquired infections divided by the number of days at risk for these infections. Prior antibiotic treatment was defined as any antibiotic treatment during the four weeks preceding ICU-admission. MDR bacteria were defined as methicillin-resistant *Staphylococcus aureus*, ceftazidime or imipenem-resistant *Pseudomonas aeruginosa*, *Acinetobacter baumannii *and *Stenotrophomonas maltophilia *and extending spectrum β-lactamase producing Gram-negative bacilli [19]. During the study period, no vancomycin-resistant *Enterococcus *was isolated in the ICU.

### Statistical methods

SPSS 11.5 software (SPSS, Chicago, IL, USA) was used for data analysis. Results are presented as number (percentage) for categorical variables, and mean ± standard deviation for quantitative variables. Distribution of quantitative variables was tested. All *P *values were two-tailed. The statistical significance was defined as *P *< 0.05.

Univariate analysis was used to determine factors associated with ICU-acquired infection. All the above cited variables were included in this analysis. Qualitative variables were compared using the Pearson chi-square test or the Fisher's exact test, as appropriate. Quantitative variables were compared using the Mann-Whitney U test or the Student's t-test, as appropriate. In patients with ICU-acquired infection, exposure to potential risk factors was taken into account until occurrence of the last ICU-acquired infection. For example, remifentanil discontinuation was considered a risk factor if it occurred before ICU-acquired infection. In patients without ICU-acquired infection, exposure to potential risk factors was taken into account until ICU discharge. Similarly, length of hospital stay was taken into account until ICU discharge in patients without ICU-acquired infection, and until the last ICU-acquired infection in patients with ICU-acquired infection. Patients with several ICU-acquired infections were considered at risk until the occurrence of the last infection.

Multivariate analysis was used to determine factors independently associated with ICU-acquired infection. All predictors showing a *P *< 0.1 association with ICU-acquired infection in univariate analysis were incorporated in the multivariate logistic regression analysis. Potential interactions were tested. Odds ratio (OR) and 95% confidence interval (CI) were calculated, as well as the Hosmer-Lemshow goodness-of-fit.

In the subgroup of patients who received remifentanil for at least 96 hours before discontinuation, incidence rate of ICU-acquired infection, duration of antimicrobial treatment and rate of patients with antimicrobial treatment discontinuation were compared between the two periods of 96 hours before and after remifentanil discontinuation. Further, incidence rate of ICU-acquired infection was compared between the 96 hours following remifentanil discontinuation and the whole ICU stay. Patients who died before remifentanil discontinuation were excluded from this analysis. For these comparisons, paired student's t test, and McNemar's test were used for quantitative and categorical variables, respectively. In this subgroup, characteristics of patients with or without ICU-acquired infection were compared at ICU admission.

## Results

During the study period, 587 patients were hospitalised in the ICU for more than 48 hours, and were all included in the study. Patient characteristics at ICU admission, and during ICU stay are presented in Table 1 and Table 2, respectively.

**Table 1:** Characteristics of study patients at intensive care unit admission

**Characteristic**	**ICU-acquired infection** **n = 233**	**No ICU-acquired infection** **n = 354**	***P *value**
Age	61 ± 14	57 ± 16	< 0.001
Male gender	161 (69)	244 (68)	> 0.999
SAPS II	54 ± 17	51 ± 20	< 0.001
LOD score	5.8 ± 3.6	4.6 ± 3.6	< 0.001
McCabe score			0.006
Nonfatal underlying disease	95 (40)	181 (51)	
Ultimately fatal underlying disease	100 (42)	142 (40)	
Rapidly fatal underlying disease	38 (16)	31 (8)	
Glasgow coma score	11 ± 2	12 ± 2	0.651
Admission category			0.129
Medical	161 (69)	266 (75)	
Surgical	72 (30)	88 (24)	
Transfer from other wards	166 (71)	198 (55)	< 0.001*
Comorbidities			
COPD	63 (27)	101 (28)	0.708
Chronic heart failure	55 (23)	72 (20)	0.358
Immunosuppression	60 (25)	68 (19)	0.066
Diabetes mellitus	34 (14)	78 (22)	0.025†
Infection	156 (66)	204 (57)	0.025‡
Prior antibiotic treatment	112 (48)	151 (42)	0.204
Length of prior hospital stay, days, median (interquartile range)	2 (0 to 6)	1 (0 to 3)	< 0.001

Data are presented as mean ± standard deviation or number (%), unless otherwise specified.

*Odds ratio = 1.95, 95% confidence interval = 1.37 to 2.77; †Odds ratio = 0.60, 95% confidence interval = 0.38 to 0.94; ‡Odds ratio = 1.49, 95% confidence interval = 1.05 to 2.10.

COPD = chronic obstructive pulmonary disease; ICU = intensive care unit; LOD = logistic organ dysfunction; SAPS = simplified acute physiology score.

**Table 2:** Characteristics of study patients during intensive care unit stay

**Characteristic**	**ICU-acquired infection** **n = 233**	**No ICU-acquired infection** **n = 354**	***P *value**	**OR (95% CI)**
Central venous catheter	224 (96)	246 (69)	< 0.001	10.9 (5.40 to 22.08)
Duration of central venous catheter use, days	25 ± 19	13 ± 9	< 0.001	
Arterial catheter	218 (93)	218 (61)	< 0.001	9.06 (5.15 to 15.9)
Duration of arterial catheter use, days	25 ± 19	12 ± 7	< 0.001	
Urinary catheter	223 (95)	281 (79)	< 0.001	5.79 (2.92 to 11.47)
Duration of urinary catheter use, days	25 ± 19	13 ± 9	< 0.001	
Mechanical ventilation	225 (96)	244 (68)	< 0.001	12.6 (6.04 to 26.5)
Duration of mechanical ventilation, days	24 ± 18	11 ± 7	< 0.001	
Reintubation	33 (14)	18 (5)	< 0.001	3.08 (1.69 to 5.61)
Tracheostomy	55 (23)	24 (6)	< 0.001	4.24 (2.54 to 7.09)
Fibreoptic bronchoscopy	140 (60)	93 (26)	< 0.001	4.22 (2.96 to 6.01)
Digestive tract endoscopy	52 (22)	32 (9)	< 0.001	2.89 (1.79 to 4.65)
Antimicrobial treatment	211 (90)	286 (80)	0.001	2.28 (1.36 to 3.80)
Duration of antimicrobial treatment, days	19 ± 16	11 ± 6	< 0.001	
Remifentanil use	203 (87)	191 (53)	< 0.001	5.77 (3.73 to 8.93)
Duration of remifentanil use, days	12 ± 11	7 ± 4	< 0.001	
Dose of remifentanil, mg/kg/day	0.73 ± 0.25	0.61 ± 0.21	0.083	
Remifentanil discontinuation	153 (65)	133 (37)	< 0.001	3.17 (2.24 to 4.49)
Midazolam use	136 (58)	106 (29)	< 0.001	3.28 (2.32 to 4.63)
Duration of midazolam use, days	9 ± 7	5 ± 4	< 0.001	
Dose of midazolam, mg/kg/days	1.76 ± 0.91	1.36 ± 0.70	0.183	
Midazolam discontinuation	86 (36)	73 (20)	< 0.001	2.25 (1.55 to 3.26)
Ramsay score	3.6 ± 1	2.7 ± 1	0.043	
Neuromuscular blocking agent use	36 (15)	25 (7)	0.001	2.40 (1.40 to 4.12)
Acute withdrawal	48 (20)	32 (9)	< 0.001	2.61 (1.61 to 4.23)
Length of stay before ICU-acquired infection, days	24 ± 19	11 ± 8	< 0.001	

Data are presented as mean ± SD or number (%).

In patients with ICU-acquired infection, exposure to potential risk factors was taken into account until occurrence of the last ICU-acquired infection. In patients without ICU-acquired infection, exposure to potential risk factors was taken into account until ICU-discharge.

ICU, intensive care unit; OR = odds ratio; SD = standard deviation.

In 233 (39%) patients, 477 microbiologically confirmed ICU-acquired infections were diagnosed. Incidence rate of ICU-acquired infection was 38 per1000 ICU days. VAP was the most frequently diagnosed ICU-acquired infection (17 per 1000 mechanical ventilation days), followed by ICU-acquired BSI (9 per 1000 ICU days), ICU-acquired urinary tract infection (8 per 1000 urinary catheter days), ventilator-associated tracheobronchitis (8 per 1000 mechanical ventilation days), catheter-related infection (2 per 1000 catheter days) and other infections (1 per 1000 ICU days). Of 233 patients with ICU-acquired infection, 198 (84%) had at least one episode of VAP or ICU-acquired BSI. *P. aeruginosa *was the most frequently isolated bacteria (30%), followed by *Enterobacter *species (13%) and *S. aureus *(10%). Twenty-eight (5%) ICU-acquired infections were polymicrobial and 151 (33%) ICU-acquired infections were related to MDR bacteria. Sixty-eight (14%) ICU-acquired infections were early onset, and mean time from ICU admission to first ICU-acquired infection was 11 ± 8 days.

Among the 394 patients who received sedation during the ICU stay, 90 patients died before remifentanil discontinuation. Remifentanil was discontinued in 304 patients, including 286 patients in which remifentanil was discontinued before ICU-acquired infection and 18 patients in which remifentanil was discontinued after ICU-acquired infection. No significant difference was found in the reintubation rate between patients with remifentanil discontinuation and patients without remifentanil discontinuation (29 of 304 (9%) vs 22 of 283 (7%), *P *= 0.242). Mean time from remifentanil discontinuation to subsequent ICU-acquired infection was 4 ± 3 days.

### Risk factors for ICU-acquired infection

Several factors were significantly associated with ICU-acquired infection by univariate analysis at ICU-admission and during ICU stay (Tables 1 and 2).

Multivariate analysis identified remifentanil discontinuation, SAPS II at ICU admission, mechanical ventilation, tracheostomy, central venous catheter and length of hospital stay as independent risk factors for ICU-acquired infection (Table 3).

**Table 3:** Risk factors for ICU-acquired infection by multivariate analysis

	OR	95% CI	*P*
Remifentanil discontinuation	2.53	1.28 to 4.99	0.007
SAPS II at ICU admission	1.01*	1 to 1.03	0.011
Mechanical ventilation	4.49	1.52 to 13.2	0.006
Tracheostomy	2.25	1.13 to 4.48	0.021
Central venous catheter	2.9	1.08 to 7.74	0.033
Length of hospital stay	1.05*	1.03 to 1.08	< 0.001

*Per point of SAPS II, and per day; respectively.

Lemeshow goodness-of-fit test, *P *= 0.86.

CI = confidence interval; ICU = intensive care unit; OR = odds ratio; SAPS = simplified acute physiology score.

### Outcomes

Duration of mechanical ventilation (33 ± 23 vs 11 ± 7 days, *P *< 0.001), length of ICU stay (38 ± 30 vs 14 ± 9 days, *P *< 0.001) and ICU mortality (108 of 233 (46%) vs 90 of 354 (25%), *P *< 0.001, OR = 2.53, 95% CI = 1.78 to 3.60) were significantly higher in patients with ICU-acquired infection compared with patients without ICU-acquired infection, respectively.

### Incidence rate of ICU-acquired infection before and after remifentanil discontinuation

**Figure 1:**
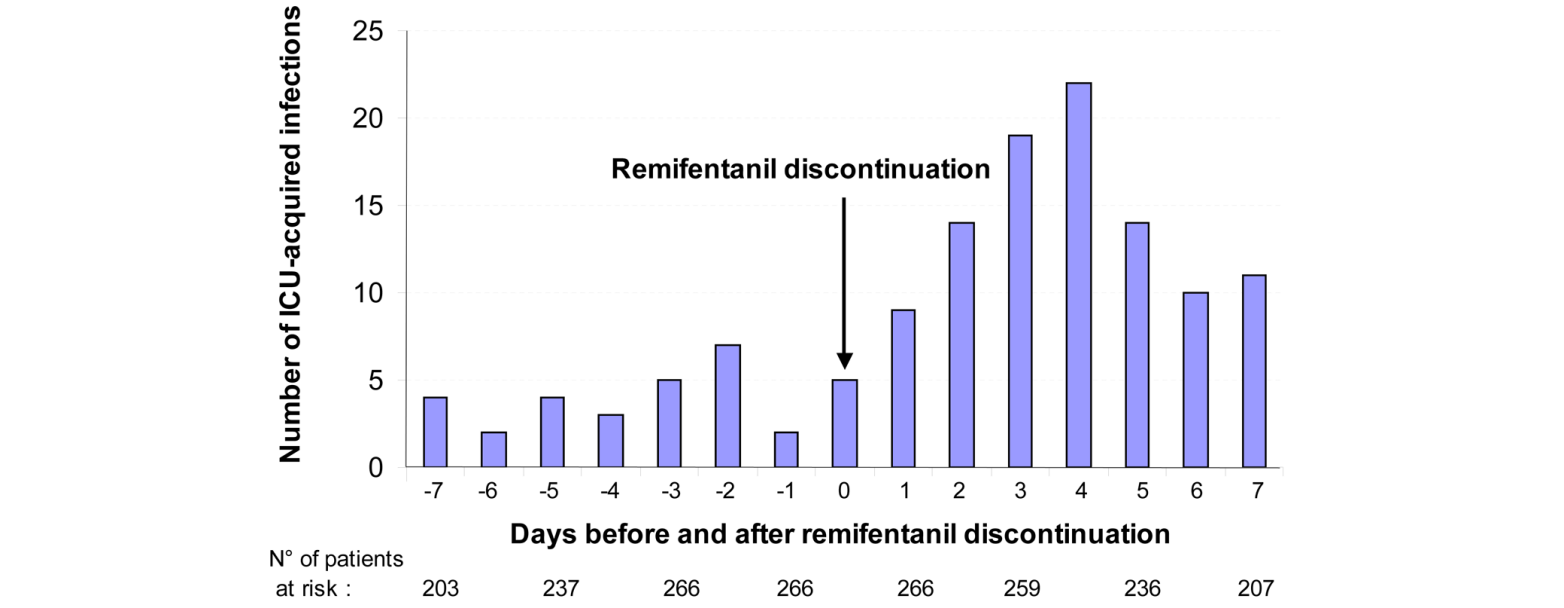
Distribution of ICU-acquired infections according to remifentanil discontinuation in patients who received remifentanil for 96 hours of more before discontinuation. Mean (standard deviation) length of intensive care unit (ICU) stay was 29 ± 28 days, including 17 ± 7 days before the first ICU-acquired infection.

Characteristics of the subgroup of patients who received remifentanil for at least 96 hours before discontinuation (n = 266) are presented in Table 4. In this subgroup, incidence rate of ICU-acquired infection was significantly higher during the 96 hours following remifentanil discontinuation compared with the 96 hours preceding remifentanil discontinuation (94 ± 192 vs 29 ± 103 per 1000 ICU days, *P *< 0.001) and compared with the whole ICU stay (94 ± 192 vs 40 ± 46 per 1000 ICU days, *P *< 0.001). In these patients, the highest rate of ICU-acquired infection was observed on day four after remifentanil discontinuation (Figure 1). Duration of antimicrobial treatment (3 ± 2 vs 2 ± 1 days, *P *= 0.182) and antimicrobial discontinuation rate (51 of 266 (19%) vs 69 of 266 (25%), *P *= 0.121) were similar during the two periods of 96 hours before and after remifentanil discontinuation, respectively.

**Table 4:** Characteristics at ICU admission of patients who received remifentanil for 96 hours or more before discontinuation

**Characteristic**	**ICU-acquired infection** **n = 153**	**No ICU-acquired infection** **n = 113**	***P *value**
Age	61 ± 14	53 ± 16	< 0.001
Male gender	100 (65)	78 (69)	0.620
SAPS II	51 ± 16	47 ± 19	0.035
LOD score	5.5 ± 3.4	5.2 ± 3.3	0.435
McCabe score			0.028
Nonfatal underlying disease	67 (43)	67 (59)	
Ultimately fatal underlying disease	69 (45)	40 (35)	
Rapidly fatal underlying disease	17 (11)	6 (5)	
Glasgow coma score	10 ± 2	11 ± 2	0.651
Admission category			> 0.999
Medical	110 (71)	82 (72)	
Surgical	43 (28)	31 (27)	
Transfer from other wards	107 (69)	63 (55)	< 0.024*
Comorbidities			
COPD	42 (27)	38 (33)	0.342
Chronic heart failure	37 (24)	20 (17)	0.262
Immunosuppression	34 (22)	20 (17)	0.452
Diabetes mellitus	24 (15)	30 (26)	0.043†
Infection	103 (67)	73 (64)	0.740
Prior antibiotic treatment	76 (49)	51 (45)	0.543
Length of prior hospital stay, days (interquartile range)	1 (0 to 2)	1 (0 to 5)	0.737

Data are presented as mean ± standard deviation or number (%), unless otherwise specified.

* Odds ratio = 1.84, 95% confidence interval = 1.11 to 3.06; † Odds ratio = 0.51, 95% confidence interval = 0.28 to 0.94.

COPD = chronic obstructive pulmonary disease; ICU = intensive care unit; LOD = logistic organ dysfunction; SAPS = simplified acute physiology score.

## Discussion

Multivariate analysis identified remifentanil discontinuation, SAPS II at ICU admission, use of mechanical ventilation, tracheostomy, central venous catheter and length of hospital stay as independent risk factors for ICU-acquired infection. All these factors, except remifentanil discontinuation, were identified by previous studies as important risk factors for health care-associated infections [2,23-26]. To our knowledge, our study is the first clinical study to evaluate the impact of opioid discontinuation on ICU-acquired infection [27].

Several potential explanations could be provided for the association between remifentanil discontinuation and ICU-acquired infection. First, higher rates of reintubation could be observed after discontinuation of sedation. Reintubation is a well-known risk factor for aspiration and VAP [28]. However, no significant difference was found in reintubation rate between patients with remifentanil discontinuation compared with patients without remifentanil discontinuation. In addition, reintubation was not independently associated with ICU-acquired infection. Second, antibiotic discontinuation may have influenced our results. Several studies demonstrated that systemic antibiotic treatment was associated with reduced rates of early-onset ICU-acquired infections [29,30]. On the other hand, other studies demonstrated that antibiotic treatment was a risk factor for subsequent infections related to MDR bacteria [31,32]. However, in the subgroup of patients sedated for at least 96 hours before discontinuation, duration of antimicrobial treatment and rate of patients with antimicrobial treatment discontinuation were similar during the two periods of 96 hours preceding and following remifentanil discontinuation. Third, the higher rate of ICU-acquired infection after remifentanil discontinuation could be related to immunosuppressive effects observed after opioid withdrawal.

Previous animal studies found morphine withdrawal to be associated with higher rates of infection. In mice exposed to morphine for 96 hours, the effect of morphine withdrawal on spontaneous sepsis and on oral infection with *Salmonella enterica *was recently examined [13]. Withdrawal significantly increased the *Salmonella *burden in various tissues of infected mice compared with animals who had placebo withdrawn and decreased the mean survival time. Elevated levels of proinflammatory cytokines were observed in the spleens of mice who had morphine withdrawn, compared with mice who had placebo withdrawn. The same authors [12] demonstrated a correlation between the suppression of IL-12 production and an increased susceptibility to *Salmonella *infection in mice undergoing withdrawal from morphine. Further, in another animal study, morphine withdrawal sensitised the animals to lipopolysaccharide lethality via increased production of TNF-α and nitric oxide [33].

In rats, immunomodulatory effects of morphine withdrawal were investigated alone and in the presence of the α-2-adrenergic agonist, clonidine [34]. Weight change was observed with peak decreases in weight occurring 24 hours after withdrawal. Rats withdrawn from morphine also exhibited a time-dependent suppression of immune status with significantly altered proliferation of T-cells stimulated by concanavalin A, altered proliferation of splenic T-cells stimulated by toxic shock syndrome toxin-1, altered production of the interferon-γ by concanavalin A-stimulated splenocytes and significantly altered natural-killer cell activity. These immunomodulatory effects were most evident 12 hours following morphine withdrawal. In addition, clonidine prevented withdrawal-induced immunosuppression. Other recent animal studies [35,10] confirmed these results and suggested that abrupt cessation of morphine administration resulted in an activation of stress-related pathways that may contribute to an increased susceptibility of infection during the initial withdrawal phase. These suppressive effects on the immune system were significant for up to 72 hours after withdrawal from chronic morphine. Our results are consistent with these findings because a peak of ICU-acquired infection was observed at day four after remifentanil discontinuation. Disturbances of the stress axis were reported to have a major impact on infections, including higher rates of postoperative pneumonia in long-term alcoholics [36]. A recent randomised controlled study, performed in long-term alcoholics, suggested that intervention at the level of the hypothalamus-pituitary-adrenal axis altered the immune response to surgical stress [37]. This resulted in decreased post-operative pneumonia rates and shortened ICU stay in these patients.

There are biological, as well as pathological, interactions between neuropeptide substance P, which is a modulator of neuroimmunoregulation, and opiates [38]. In an *in vitro *model, Wang and colleagues [39] investigated the relationship between morphine withdrawal and HIV infection of human T lymphocytes. They concluded that the interaction of opiates and neuropeptide substance P in human T lymphocytes was likely to a have a role in the immunopathogenesis of HIV disease among opiate abusers.

Prevalence of ICU-acquired infection and duration of sedation were high in our study. This could be explained by the high severity of our patients at ICU admission, the large proportion of patients with comorbidities and the high proportion of patients requiring mechanical ventilation. These factors are well known to be associated with prolonged ICU stay and higher risk for ICU-acquired infection [19]. A recent study was performed in 151 ICUs in France, including 30% of university ICUs [40]. During a six-month period, 20,632 patients were included in the study. Incidence rates of ICU-acquired infection were consistent with our findings, including 17.5 VAP cases per 1000 mechanical ventilation days, 2.2 catheter-related infections per 1000 catheter days, 3.3 BSI per 1000 ICU days and 7.8 urinary tract infections per 1000 urinary catheter days. Some of the ICU-acquired infections evaluated by our study could be difficult to differentiate from colonisation such as ventilator-associated tracheobronchitis and urinary tract infection. To adjust for this potential confounder, we have repeated all analyses taking into account only ICU-acquired BSI and VAP. Similar results were found (data not shown), and remifentanil discontinuation was still independently associated with ICU-acquired BSI and VAP.

It could be argued that the higher incidence rate of ICU-acquired infection during the 96 hours following sedation discontinuation, found by our study, could simply reflect the fact that the cumulative risk of ICU-acquired infection was higher during this period compared with the 96 hours preceding sedation discontinuation. However, although the cumulative risk for ICU-acquired infection was higher during the whole ICU stay compared with the 96 hours following sedation discontinuation, incidence rate of ICU-acquired infection was higher during the 96 hours following sedation discontinuation compared with the whole ICU stay. Additionally, remifentanil discontinuation was independently associated with ICU-acquired infection.

Daily sedation interruption was not performed in this study. However, a nurse-adjusted sedation protocol was used. A recent randomised controlled study compared nursing-implemented sedation algorithm with daily interruption of sedation [41]. The authors found the sedation algorithm to be associated with reduced duration of mechanical ventilation and lengths of stay compared with daily interruption of sedation. In addition, a recent web-based survey was performed on members of the Society of Critical Care Medicine [42]. Daily interruption of sedation was only performed by 40% of the 904 responders. Another recent survey demonstrated that none of the 44 participating ICUs were conducting daily interruption of sedation [43].

Our study has some limitations. First, this was a single centre study and the results may not be generalisable to other ICUs. Second, our study was observational, and further randomised studies are needed to confirm these results. Third, remifentanil was used for sedation. Whether our results are applicable to patients sedated with other opioids is unknown. A recent *in vitro *study found remifentanil, but not sufentanil or alfentanil, to attenuate lipopolysaccharide-induced neutrophil responses and neutrophil-mediated inflammatory responses [44]. Fourth, midazolam was used in a large proportion of patients sedated with remifentanil. Therefore, midazolam discontinuation may have influenced the subsequent ICU-acquired infection. However, midazolam use and discontinuation were not independently associated with ICU-acquired infection. Fifth, our risk analysis did not account for the patient's condition on the day of remifentanil discontinuation. However, a different study design, such as a matched-controlled study, would be more appropriate to adjust for this potential confounder. Finally, although remifentanil discontinuation was an independent risk factor for ICU-acquired infection, acute withdrawal was not independently associated with ICU-acquired infection. One potential explanation is the fact that our definition of acute withdrawal was stringent resulting in possible underestimation of acute withdrawal frequency. However, no relationship between infection and clinical signs of abstinence has been reported by animal studies [12,13].

## Conclusions

We conclude that remifentanil discontinuation is associated with a higher risk of ICU-acquired infections. Further randomised studies are needed to confirm these results.

## Key messages

• Remifentanil discontinuation is independently associated with a higher risk for ICU-acquired infections.

• Further randomised studies are needed to confirm these results.

## Abbreviations

BSI: bloodstream infection; cfu: colony forming units; CI: confidence interval; ICU: intensive care unit; IL: interleukin; MDR: multidrug-resistant; OR: odds ratio; SAPS: simplified acute physiology score; TNF: tumour necrosis factor; VAP: ventilator-associated pneumonia.

## Competing interests

The authors declare that they have no competing interests.

## Authors' contributions

SN, DM and AD designed the study. SN, JH, GG and ASL collected the data. CDP performed statistical analyses. SN wrote the manuscript, and all authors participated in its critical revision. SN had full access to all data in the study and had final responsibility for the decision to submit for publication. All authors read and approved the final manuscript.

## References

[B1] Burke JP (2003). Infection control – a problem for patient safety. N Engl J Med.

[B2] Hugonnet S, Chevrolet JC, Pittet D (2007). The effect of workload on infection risk in critically ill patients. Crit Care Med.

[B3] Malacarne P, Langer M, Nascimben E, Moro ML, Giudici D, Lampati L, Bertolini G (2008). Building a continuous multicenter infection surveillance system in the intensive care unit: findings from the initial data set of 9,493 patients from 71 Italian intensive care units. Crit Care Med.

[B4] Vincent JL, Sakr Y, Sprung CL, Ranieri VM, Reinhart K, Gerlach H, Moreno R, Carlet J, Le Gall JR, Payen D (2006). Sepsis in European intensive care units: results of the SOAP study. Crit Care Med.

[B5] Safdar N, Abad C (2008). Educational interventions for prevention of healthcare-associated infection: a systematic review. Crit Care Med.

[B6] Nseir S, Di Pompeo C, Soubrier S, Cavestri B, Jozefowicz E, Saulnier F, Durocher A (2005). Impact of ventilator-associated pneumonia on outcome in patients with COPD. Chest.

[B7] Jacobi J, Fraser GL, Coursin DB, Riker RR, Fontaine D, Wittbrodt ET, Chalfin DB, Masica MF, Bjerke HS, Coplin WM, Crippen DW, Fuchs BD, Kelleher RM, Marik PE, Nasraway SA, Murray MJ, Peruzzi WT, Lumb PD (2002). Clinical practice guidelines for the sustained use of sedatives and analgesics in the critically ill adult. Crit Care Med.

[B8] Muellejans B, Matthey T, Scholpp J, Schill M (2006). Sedation in the intensive care unit with remifentanil/propofol versus midazolam/fentanyl: a randomised, open-label, pharmacoeconomic trial. Crit Care.

[B9] Wilhelm W, Kreuer S (2008). The place for short-acting opioids: special emphasis on remifentanil. Crit Care.

[B10] Rahim RT, Adler MW, Meissler JJ, Cowan A, Rogers TJ, Geller EB, Eisenstein TK (2002). Abrupt or precipitated withdrawal from morphine induces immunosuppression. J Neuroimmunol.

[B11] Govitrapong P, Suttitum T, Kotchabhakdi N, Uneklabh T (1998). Alterations of immune functions in heroin addicts and heroin withdrawal subjects. J Pharmacol Exp Ther.

[B12] Feng P, Wilson QM, Meissler JJ, Adler MW, Eisenstein TK (2005). Increased sensitivity to Salmonella enterica serovar Typhimurium infection in mice undergoing withdrawal from morphine is associated with suppression of interleukin-12. Infect Immun.

[B13] Feng P, Truant AL, Meissler JJ, Gaughan JP, Adler MW, Eisenstein TK (2006). Morphine withdrawal lowers host defense to enteric bacteria: spontaneous sepsis and increased sensitivity to oral Salmonella enterica serovar Typhimurium infection. Infect Immun.

[B14] Nseir S, Di Pompeo C, Diarra M, Brisson H, Tissier S, Boulo M, Durocher A (2007). Relationship between immunosuppression and intensive care unit-acquired multidrug-resistant bacteria: a case-control study. Crit Care Med.

[B15] Ramsay MA, Savege TM, Simpson BR, Goodwin R (1974). Controlled sedation with alphaxalone-alphadolone. BMJ.

[B16] Le Gall JR, Lemeshow S, Saulnier F (1993). A new Simplified Acute Physiology Score (SAPS II) based on a European/North American multicenter study. JAMA.

[B17] Celli BR, MacNee W (2004). Standards for the diagnosis and treatment of patients with COPD: a summary of the ATS/ERS position paper. Eur Respir J.

[B18] Korak-Leiter M, Likar R, Oher M, Trampitsch E, Ziervogel G, Levy JV, Freye EC (2005). Withdrawal following sufentanil/propofol and sufentanil/midazolam. Sedation in surgical ICU patients: correlation with central nervous parameters and endogenous opioids. Intensive Care Med.

[B19] Niederman MS, Craven D (2005). Guidelines for the management of adults with hospital-acquired, ventilator-associated, and healthcare-associated pneumonia. Am J Respir Crit Care Med.

[B20] Martin MA, Pfaller MA, Wenzel RP (1989). Coagulase-negative staphylococcal bacteremia. Mortality and hospital stay. Ann Intern Med.

[B21] Nseir S, Favory R, Jozefowicz E, Decamps F, Dewavrin F, Brunin G, Di Pompeo C, Mathieu D, Durocher A (2008). Antimicrobial treatment for ventilator-associated tracheobronchitis: a randomized, controlled, multicenter study. Crit Care.

[B22] Garner JS, Jarvis WR, Emori TG, Horan TC, Hughes JM (1988). CDC definitions for nosocomial infections, 1988. Am J Infect Control.

[B23] Kooi TI van der, de Boer AS, Mannien J, Wille JC, Beaumont MT, Mooi BW, van den HS (2007). Incidence and risk factors of device-associated infections and associated mortality at the intensive care in the Dutch surveillance system. Intensive Care Med.

[B24] Koh DB, Gowardman JR, Rickard CM, Robertson IK, Brown A (2008). Prospective study of peripheral arterial catheter infection and comparison with concurrently sited central venous catheters. Crit Care Med.

[B25] Alp E, Guven M, Yildiz O, Aygen B, Voss A, Doganay M (2004). Incidence, risk factors and mortality of nosocomial pneumonia in intensive care units: a prospective study. Ann Clin Microbiol Antimicrob.

[B26] Apostolopoulou E, Bakakos P, Katostaras T, Gregorakos L (2003). Incidence and risk factors for ventilator-associated pneumonia in 4 multidisciplinary intensive care units in Athens, Greece. Respir Care.

[B27] Weinert CR, Kethireddy S, Roy S (2008). Opioids and Infections in the Intensive Care Unit Should Clinicians and Patients be Concerned?. J Neuroimmune Pharmacol.

[B28] Craven DE (2006). Preventing ventilator-associated pneumonia in adults: sowing seeds of change. Chest.

[B29] Acquarolo A, Urli T, Perone G, Giannotti C, Candiani A, Latronico N (2005). Antibiotic prophylaxis of early onset pneumonia in critically ill comatose patients. A randomized study. Intensive Care Med.

[B30] Sirvent JM, Torres A, El Ebiary M, Castro P, de Batlle J, Bonet A (1997). Protective effect of intravenously administered cefuroxime against nosocomial pneumonia in patients with structural coma. Am J Respir Crit Care Med.

[B31] Nseir S, Di Pompeo C, Jozefowicz E, Cavestri B, Brisson H, Nyunga M, Soubrier S, Durocher A (2007). Relationship between tracheotomy and ventilator-associated pneumonia: a case control study. Eur Respir J.

[B32] Nseir S, Di Pompeo C, Brisson H, Dewavrin F, Tissier S, Diarra M, Boulo M, Durocher A (2006). Intensive care unit-acquired Stenotrophomonas maltophilia: incidence, risk factors, and outcome. Crit Care.

[B33] Feng P, Meissler JJ, Adler MW, Eisenstein TK (2005). Morphine withdrawal sensitizes mice to lipopolysaccharide: elevated TNF-alpha and nitric oxide with decreased IL-12. J Neuroimmunol.

[B34] West JP, Dykstra LA, Lysle DT (1999). Immunomodulatory effects of morphine withdrawal in the rat are time dependent and reversible by clonidine. Psychopharmacology (Berl).

[B35] Avila AH, Alonzo NC, Bayer BM (2004). Immune cell activity during the initial stages of withdrawal from chronic exposure to cocaine or morphine. J Neuroimmunol.

[B36] Spies CD, von DV, Eggers V, Jetschmann G, El Hilali R, Egert J, Fischer M, Schroder T, Hoflich C, Sinha P, Paschen C, Mirsalim P, Brunsch R, Hopf J, Marks C, Wernecke KD, Pragst F, Ehrenreich H, Muller C, Tonnesen H, Oelkers W, Rohde W, Stein C, Kox WJ (2004). Altered cell-mediated immunity and increased postoperative infection rate in long-term alcoholic patients. Anesthesiology.

[B37] Spies C, Eggers V, Szabo G, Lau A, von DV, Schoenfeld H, Althoff H, Hegenscheid K, Bohm B, Schroeder T, Pfeiffer S, Ziemer S, Paschen C, Klein M, Marks C, Miller P, Sander M, Wernecke KD, Achterberg E, Kaisers U, Volk HD (2006). Intervention at the level of the neuroendocrine-immune axis and postoperative pneumonia rate in long-term alcoholics. Am J Respir Crit Care Med.

[B38] Tiong GK, Pierce TL, Olley JE (1992). Sub-chronic exposure to opiates in the rat: effects on brain levels of substance P and calcitonin gene-related peptide during dependence and withdrawal. J Neurosci Res.

[B39] Wang X, Douglas SD, Peng JS, Zhou DJ, Wan Q, Ho WZ (2006). An in vitro model of morphine withdrawal manifests the enhancing effect on human immunodeficiency virus infection of human T lymphocytes through the induction of substance P. Am J Pathol.

[B40] REA-RAISIN 2005: Surveillance of ICU-aquired infections in adult critically ill patients in France. http://www.invs.sante.fr.

[B41] de Wit M, Gennings C, Jenvey WI, Epstein SK (2008). Randomized trial comparing daily interruption of sedation and nursing-implemented sedation algorithm in medical intensive care unit patients. Crit Care.

[B42] Tanios MA, de Wit M, Epstein SK, Devlin JW (2009). Perceived barriers to the use of sedation protocols and daily sedation interruption: A multidisciplinary survey. J Crit Care.

[B43] Payen JF, Chanques G, Mantz J, Hercule C, Auriant I, Leguillou JL, Binhas M, Genty C, Rolland C, Bosson JL (2007). Current practices in sedation and analgesia for mechanically ventilated critically ill patients: a prospective multicenter patient-based study. Anesthesiology.

[B44] Kwak S, Chung S, Li M, Bae H (2008). Remifentanil attenuates LPS-induced neutrophil activation. Crit Care Med.

